# Integrative GWAS Catalog Analysis of Proxy-Trait Intersection in Sarcopenia-Related Musculoskeletal Aging with Multi-Criteria Evidence Integration

**DOI:** 10.3390/life16081357

**Published:** 2026-08-19

**Authors:** Hung-Wen Chen, Chen-Long Chen, Yao-Jen Liang, Yen-Lin Chen

**Affiliations:** 1 Graduate Institute of Applied Science and Engineering, Fu Jen Catholic University, New Taipei City 242, Taiwan; reddchen21@gmail.com (H.-W.C.); jackychenlong@yahoo.com.tw (C.-L.C.); 2 Department of Pathology, Tri-Service General Hospital, National Defense Medical University, New Taipei City 114, Taiwan; anthonypatho@gmail.com

**Keywords:** sarcopenia, genome-wide association study, grip strength, appendicular lean mass, frailty, musculoskeletal aging, evidence integration

## Abstract

Background: Sarcopenia is a multidimensional musculoskeletal-aging phenotype defined by reduced muscle quantity together with weakness and impaired performance, but public genetic resources rarely capture sarcopenia as a single uniformly labeled phenotype. Methods: Official National Human Genome Research Institute-European Bioinformatics Institute (NHGRI-EBI) genome-wide association studies (GWAS) Catalog association files archived locally on 15 April 2026 were grouped into muscle-quantity and function-frailty domains. Exact shared rsIDs were identified, assigned to catalog representative/mapped genes, evaluated against exploratory skeletal-muscle transcriptomic context, benchmarked with trait-grouping sensitivity analyses, and ranked with a secondary multi-criteria evidence-integration framework. Results: The muscle-quantity domain contained 1580 unique single-nucleotide polymorphisms (SNPs) and the function-frailty domain contained 383 unique SNPs, with 14 exact rsIDs shared between domains. A trait-file permutation benchmark did not show that this overlap exceeded a catalog-level null expectation (empirical *p* = 0.514), so the shared set is interpreted as a descriptive, high-specificity candidate intersection rather than statistically enriched sharing. Sensitivity analyses showed that the 14-rsID set was retained after excluding falling/fall and chronic obstructive pulmonary disease (COPD)-related proxy traits, indicating that the primary shared set was driven mainly by grip/low-grip function traits. Exploratory transcriptomic context and evidence integration organized candidates for follow-up but did not validate causal or linkage disequilibrium (LD)-level sharing. Conclusions: This framework generates candidate hypotheses for sarcopenia-related musculoskeletal aging but does not establish causal, fine-mapped, or statistically enriched genetic sharing.

## 1. Introduction

Sarcopenia is increasingly recognized as a multidimensional phenotype of musculoskeletal aging rather than as an isolated reduction in muscle mass [1,2]. Its clinical significance arises from the combined effects of reduced muscle quantity, weakness, impaired physical performance, falls, disability, and adverse outcomes [3,4]. This distinction is important because older adults may show discordance between muscle mass and functional performance, and clinically meaningful impairment often emerges when structural reserve, neuromuscular capacity, and systemic vulnerability deteriorate together [5,6]. Contemporary reviews further emphasize that sarcopenia involves skeletal muscle biology, neuromuscular function, inflammation, metabolism, and broader aging-related vulnerability [7,8].

This multidimensional clinical definition creates a specific challenge for genetic analysis. Large public association resources rarely represent sarcopenia as a single, uniformly defined, well-powered phenotype; instead, relevant biology is distributed across component traits such as appendicular lean mass, hand-grip strength, muscle weakness, frailty index, falls, and related functional outcomes [9,10]. Previous genome-wide association studies (GWAS) have provided valuable information about lean-mass biology [11,12,13], grip-strength phenotypes [14,15], and additional strength-related signals from sequencing or population-specific analyses [16,17]. Other genetic studies have examined muscle weakness [18], frailty [19,20], falling risk [21,22], and cohort-specific sarcopenia definitions [23,24]. These studies define important component signals but do not by themselves establish whether the components converge on a shared sarcopenia-related architecture.

A proxy-trait framework is therefore biologically and clinically appropriate for investigating sarcopenia-related genetics in currently available resources. Longitudinal and genetic studies link grip strength with frailty, physical performance, and functional limitations [25], while sarcopenia-related traits have also been connected with falls and fall-related vulnerability [26]. Genetic and Mendelian-randomization studies have further examined metabolic determinants, osteoarthritis, and hip-fracture susceptibility in relation to sarcopenia-related or musculoskeletal traits [27,28,29]. Complementary clinical literature has proposed hand-grip strength as a broader health indicator and emphasized biomarkers and multifactorial determinants of frailty in older adults [30,31,32]. More directly relevant genetic literature includes aging-muscle genetic-association reviews, sarcopenia-specific GWAS studies, chronic obstructive pulmonary disease (COPD)-sarcopenia genetic analyses, sarcopenic-obesity exome-wide analyses, and recent reviews of sarcopenia-related single-nucleotide polymorphisms (SNPs) evidence [23,24,33,34,35,36,37]. These sources support the premise that sarcopenia-related biology is distributed across muscle-mass, strength, and vulnerability phenotypes, but they do not independently validate the present 14-rsID catalog intersection. The key premise is not that any single proxy trait is equivalent to sarcopenia, but that clinically aligned proxy domains may capture complementary dimensions of the same aging-related process.

The present study addresses this gap by reconstructing a clinically aligned proxy-trait architecture from official National Human Genome Research Institute-European Bioinformatics Institute (NHGRI-EBI) GWAS Catalog resources. Muscle-quantity traits were grouped against function-frailty traits to identify exact rsID intersections between selected catalog domains. This design focuses on sarcopenia-related musculoskeletal aging hypotheses rather than on nonspecific aging-associated GWAS signals. Because the analysis is based on exact shared rsID overlap, the resulting loci are interpreted as a high-specificity candidate subset of catalog intersections rather than as the full genetic correlation, linkage disequilibrium (LD)-mediated sharing, or causal architecture between domains.

The analytical objective was to determine whether proxy traits reflecting diagnostic components of sarcopenia contain a compact and interpretable set of shared catalog entries. To strengthen biological interpretation, the overlap-derived loci were evaluated against public skeletal-muscle transcriptomic context, allowing catalog-derived genetic signals to be considered alongside exploratory tissue-level evidence. A secondary multi-criteria evidence-integration layer was then used to organize heterogeneous evidence across recurrence, trait balance, pleiotropic breadth, transcriptomic support, and rank stability. This prioritization component was designed to support transparent candidate ranking rather than to replace conventional genetic or biological interpretation.

Together, this framework positions sarcopenia-related genetics as a problem of clinically informed signal integration. Rather than treating muscle quantity, weakness, frailty, and falls as isolated endpoints, the study evaluates whether these distributed public phenotypes recover an interpretable candidate pattern when analyzed through a shared-catalog-entry lens. The resulting analysis is intended as a hypothesis-generating resource for identifying candidate loci that may connect body composition, functional decline, and aging-related vulnerability.

## 2. Materials and Methods

### 2.1. Study Design

This study was conducted as an integrative GWAS dry-lab analysis using official NHGRI-EBI GWAS Catalog association files (https://www.ebi.ac.uk/gwas/ (accessed on 9 August 2026)). Trait files were obtained from official GWAS Catalog resources and locally grouped into two domains representing sarcopenia-related musculoskeletal aging: muscle quantity and function-frailty. The supplementary GWAS inventory lists each included source file, study accession identifiers (GCST IDs), PMIDs, association-row counts, unique-rsID counts, and initial sample-size text. Publicly available, de-identified catalog associations were used throughout, and no new human participant recruitment or intervention was performed [9,10]. The study was designed as a descriptive and hypothesis-generating screen of curated trait domains rather than as a harmonized causal GWAS, fine-mapping, or colocalization analysis.

### 2.2. Phenotype Grouping

The muscle-quantity group included appendicular lean mass, appendicular skeletal muscle mass, and lean-mass-related traits. The function-frailty group included hand-grip strength, low hand-grip strength, European Working Group on Sarcopenia in Older People (EWGSOP) and Foundation for the National Institutes of Health (FNIH) low-grip definitions, frailty index, falling risk, and limited sarcopenia-in-COPD proxy traits. Inclusion required that a GWAS Catalog trait captured structural muscle reserve or downstream functional vulnerability commonly used to operationalize sarcopenia-related impairment in aging populations. Traits were excluded from the main grouping when they primarily represented broad adiposity or nonspecific body-size phenotypes without a direct muscle-quantity or functional-vulnerability interpretation. The COPD-related proxy traits were retained only as limited secondary function-frailty proxies because COPD-associated weakness can reflect cachexia, hypoxia, systemic inflammation, and COPD-specific sarcopenia biology [33,38,39]; this choice is therefore interpreted cautiously. Group assignment was clinically motivated rather than purely ontology driven. Prespecified sensitivity checks evaluated exclusion of falling/fall traits, COPD-related proxy traits, both falling/fall and COPD-related proxy traits, a hand-grip/low-grip-only function domain, and a frailty/falls-only function domain.

### 2.3. Shared-SNP Analysis

#### 2.3.1. Gene Mapping and Functional Interpretation

Catalog representative/mapped genes were assigned primarily from the GWAS Catalog MAPPED_GENE field, with REPORTED GENE(S) used as a secondary source when mapped-gene information was unavailable or ambiguous. For entries containing multiple mapped genes, the nearest or catalog-designated gene was retained as the representative label for tabulation, while the raw mapped-gene string was preserved in the Supplementary Tables S1–S6. These labels should not be interpreted as confirmed effector genes. Intergenic, long non-coding RNA, and pseudogene assignments were not discarded, but were labeled as less-resolved because proximity-based annotation may not identify the effector gene. No external variant-to-gene algorithm, expression quantitative trait locus (eQTL) colocalization, Open Targets variant-to-gene (V2G) scoring, or Genotype-Tissue Expression (GTEx)-based reassignment was used in the primary analysis. Shared loci were organized into descriptive functional modules for interpretation and figure design rather than for formal pathway enrichment.

For each grouped domain, unique rsIDs were extracted from the GWAS Catalog association files. The two groups were intersected to identify exact shared SNPs. Source association rows corresponding to the shared SNP set were pooled for downstream locus summarization, catalog representative/mapped gene assignment, and evidence counting. Study-level overlap counts are summarized in Table 1. Exact-rsID overlap was used as a conservative, high-specificity intersection screen. This approach is deliberately narrower than LD-based locus overlap and may miss shared causal architecture when different variants tag the same locus across studies or ancestries. Conversely, exact rsID sharing can be inflated when the same participants or biobank-derived GWAS contribute to both domains. As an exploratory complement, we also summarized coordinate-window overlap using +/−500 kb around catalog rsID positions; this window-based check was not treated as LD-based colocalization. No LD clumping, ancestry-specific LD harmonization, fine-mapping, or colocalization was performed.

#### 2.3.2. Multi-Criteria Evidence-Integration Framework

An interpretable, secondary multi-criteria evidence-integration framework was applied after the core overlap analysis. Five features were min-max normalized across the 14 representative genes: total GWAS recurrence, quantity-function balance, phenome-wide association study (PheWAS) hit volume, PheWAS domain diversity, and transcriptomic support count. The weighted evidence-fusion score was calculated as 0.30 × total recurrence + 0.15 × balance + 0.20 × PheWAS hit volume + 0.20 × PheWAS domain diversity + 0.15 × transcriptomic support. A latent prioritization score was derived from the first component of singular-value decomposition applied to the normalized feature matrix and rescaled to 0–1. Rank stability was evaluated with 1000 Monte Carlo iterations in which feature weights were randomly perturbed by +/−20% and renormalized to sum to 1; the bootstrap consensus score reflects normalized rank stability and top-rank retention across iterations. The final ensemble score was calculated as 0.50 × evidence-fusion score + 0.30 × latent prioritization score + 0.20 × bootstrap consensus score. Weighting sensitivity was evaluated under equal feature weights, removal of transcriptomic support, and removal of PheWAS breadth. This framework ranks heterogeneous evidence for follow-up but should not be interpreted as an independently validated predictive model.

## 3. Results

### 3.1. Muscle Quantity and Function-Frailty Traits Share a Measurable Overlap

The muscle-quantity group contained 1580 unique SNPs, whereas the function-frailty group contained 383 unique SNPs. Fourteen SNPs were shared between the two groups (Table 1). To benchmark this overlap, we performed a trait-file permutation analysis across the 15 included GWAS Catalog source files, randomly assigning three files to a pseudo muscle-quantity domain and the remaining files to the comparison domain for all 455 possible combinations. The permutation distribution had a mean overlap of 12.25 rsIDs, a median of 14, and a range of 0–28; the empirical probability of observing at least 14 shared rsIDs was 0.514. A hypergeometric calculation using only the selected-trait union as the background was not informative because the domain-specific SNP sets occupy a large fraction of that restricted universe. Therefore, the 14 shared rsIDs are interpreted as a descriptive, high-specificity candidate intersection rather than as statistically enriched evidence of genome-wide sharing. Trait-grouping sensitivity analyses retained all 14 shared rsIDs after excluding falling/fall traits, after excluding COPD-related proxy traits, after excluding both falling/fall and COPD-related proxy traits, and in a hand-grip/low-grip-only function domain. In contrast, a frailty/falls-only function domain recovered no shared rsIDs, indicating that the primary shared set should be interpreted mainly as a muscle-quantity and grip/low-grip intersection rather than as evidence for a broad frailty/falls genetic architecture.

Null-benchmarking note: A trait-file permutation benchmark across the 15 included GWAS Catalog files produced a mean overlap of 12.25 rsIDs, median overlap of 14, and empirical *p* = 0.514 for an overlap of at least 14 rsIDs. The observed shared set is therefore treated as a descriptive candidate intersection rather than as statistically enriched sharing.

Trait-grouping sensitivity note: The same 14 shared rsIDs were retained when falling/fall traits were excluded, when COPD-related proxy traits were excluded, when both falling/fall and COPD-related proxy traits were excluded, and when the function domain was restricted to hand-grip or low-grip traits. A frailty/falls-only function domain recovered no shared rsIDs. This supports interpretation of the primary overlap as grip/low-grip driven rather than dependent on falls or COPD proxies.

### 3.2. Shared Sarcopenia-Related Musculoskeletal-Aging Loci

**Figure 1:**
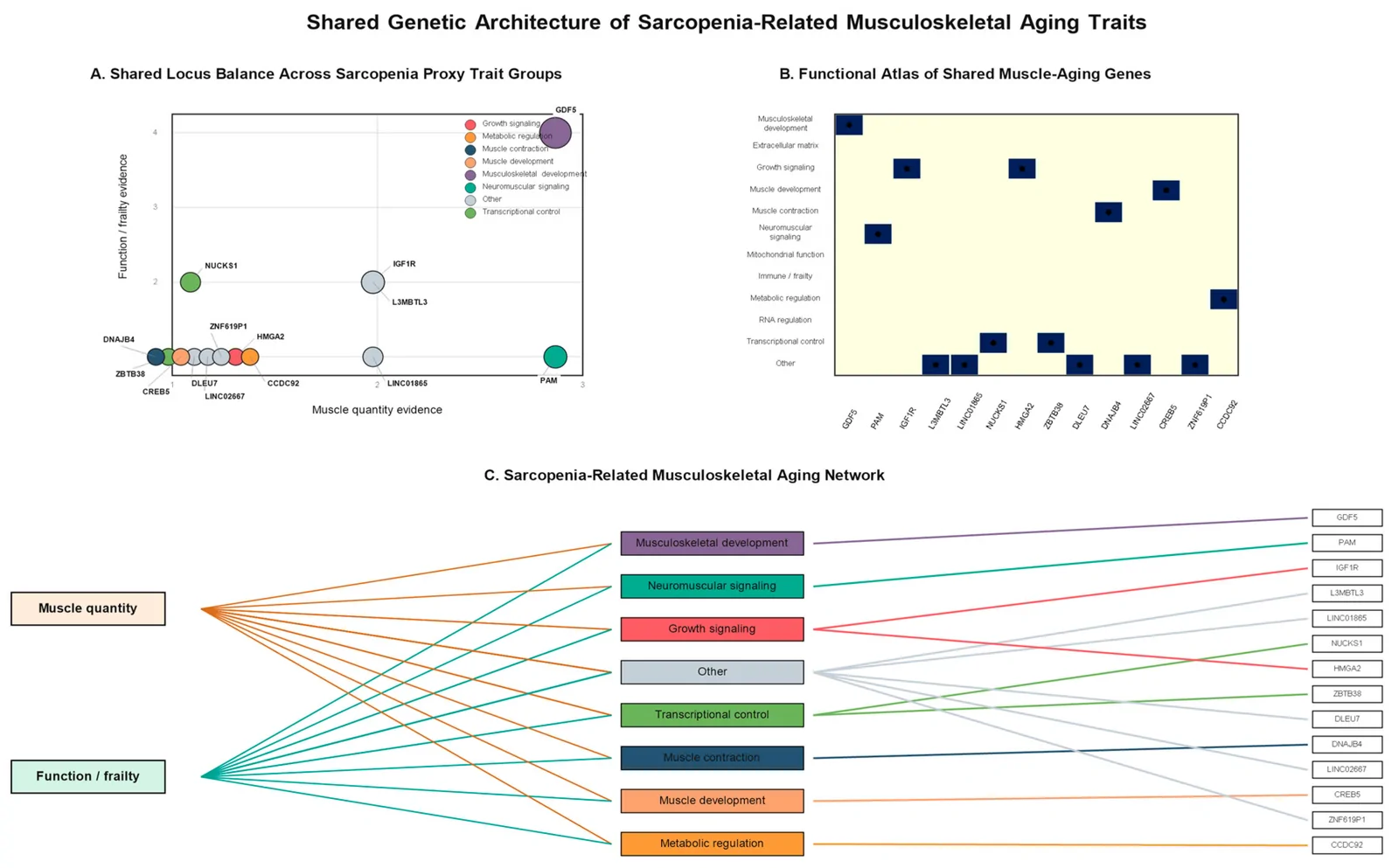
(**A**) Association-row support across the two proxy domains; bubble size indicates the number of supporting GWAS Catalog association rows, and color indicates the assigned heuristic category. (**B**) Distribution of representative shared loci across functional categories. (**C**) Descriptive map linking grouped phenotypes, heuristic categories, and representative loci; connections do not imply causality, network topology, or formal pathway enrichment.

After source-row aggregation, the shared overlap resolved into recurrent loci including *GDF5*, *PAM*, *IGF1R*, *HMGA2*, *NUCKS1*, *ZBTB38*, *DNAJB4*, *CREB5*, and *CCDC92*. *GDF5* showed the highest catalog recurrence across both groups, but this reflects the number of recovered GWAS Catalog association rows and not necessarily effect size, causal primacy, or muscle-specific mechanism. *IGF1R* and *HMGA2* reinforced a growth-related component, *PAM* and *DNAJB4* contributed neuromuscular or tissue-response signals, and *NUCKS1* and *ZBTB38* supported a regulatory layer. The domain-level overlap structure and representative locus grouping are shown in Figure 1. The full 14-locus shared output is summarized in Table 2. Together, these loci define a focused candidate pattern involving developmental patterning, tissue growth, regulatory control, and functional decline.

Figure 1 provides an overview of shared sarcopenia-related musculoskeletal aging loci.

### 3.3. Developmental and Growth-Signaling Loci Link Lean-Mass Biology to Age-Related Weakness

The presence of *GDF5*, *IGF1R*, and *HMGA2* is notable because these loci provide biologically interpretable candidates at the interface of muscle quantity and measured muscle performance. Large lean-mass GWAS have emphasized inherited contributions to muscle quantity and tissue architecture [11,12,13]. Muscle-weakness meta-analysis further supports genetic links between inherited variation and later-life low-strength phenotypes [18]. Cohort-specific sarcopenia GWAS also indicate that sarcopenia-related definitions can recover partially overlapping genetic signals [23].

### 3.4. Grouped Proxy Overlap Extends the Clinical Framing

Grip-strength and frailty studies are often analyzed separately, and the present catalog intersection should be interpreted as a candidate overlap across selected related phenotypes rather than as proof of broad frailty-driven shared genetic architecture. Frailty GWAS have identified genetic associations relevant to aging vulnerability [19,20]. Longitudinal evidence further links grip strength with frailty, physical performance, and functional limitations [25]. The inclusion of falling risk extends the clinical context from measured muscle performance to vulnerability outcomes [21,22], consistent with evidence connecting sarcopenia-related traits to falls in older adults [26].

### 3.5. Public Context for Skeletal-Muscle Tissue Biology

**Figure 2:**
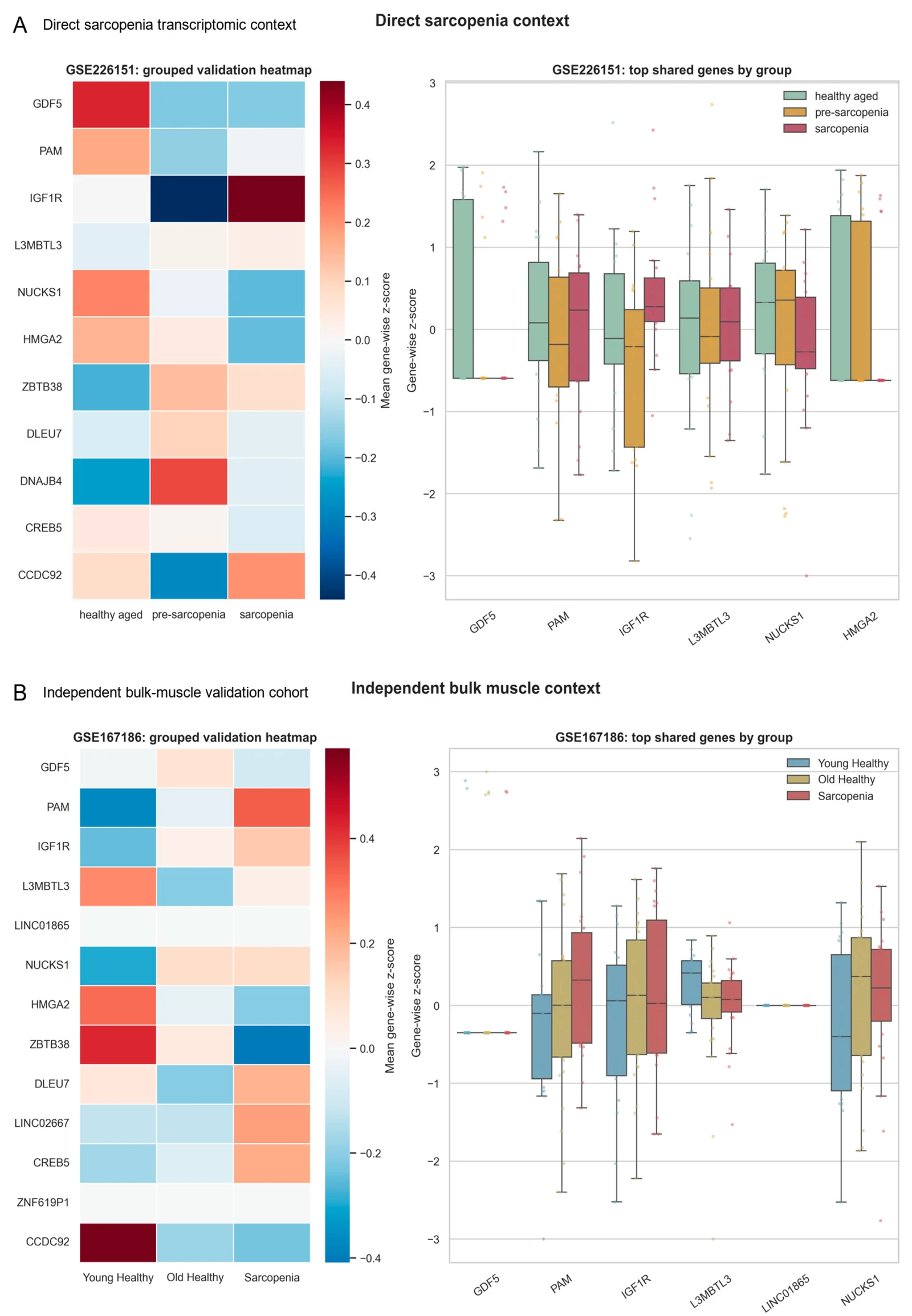
(**A**) shows the direct sarcopenia transcriptomic context in GSE226151 (*n* = 60) across healthy aged (*n* = 20), pre-sarcopenia (*n* = 20), and sarcopenia (*n* = 20) muscle samples. (**B**) shows the independent bulk skeletal-muscle contextualization cohort GSE167186 (*n* = 72) across young healthy (*n* = 19), old healthy (*n* = 29), and sarcopenia (*n* = 24) groups. These panels evaluate whether overlap-derived loci are detectable across disease progression and an independent cohort structure; they do not constitute formal transcriptomic validation.

Public skeletal-muscle transcriptomic context was used as supportive contextualization rather than formal validation. The public transcriptomic layer used retained Gene Expression Omnibus (GEO) count, transcripts per million (TPM), Simple Omnibus Format in Text (SOFT), series-matrix, or processed summary files; raw FASTQ-level reprocessing was not performed. Analyses were implemented in retained Python (v3.14.4) scripts using pandas (v3.0.3), NumPy (v2.5.1), SciPy (1.18.0), matplotlib (v3.11.1), and seaborn (v0.13.2). In GSE226151 (*n* = 60), a direct sarcopenia cohort, grouped expression patterns were evaluated across healthy-aged (*n* = 20), pre-sarcopenia (*n* = 20), and sarcopenia (*n* = 20) muscle samples. In GSE167186 (*n* = 72), an independent bulk-muscle cohort, *PAM* increased in sarcopenia whereas *ZBTB38* and *CCDC92* decreased relative to young healthy muscle using nominal unadjusted endpoint tests across young healthy (*n* = 19), old healthy (*n* = 29), and sarcopenia (*n* = 24) groups. In GSE164471 (*n* = 53), *PAM* also increased with age across healthy muscle using Spearman trend testing; age-bin counts were 20–49 years (*n* = 23), 50–64 years (*n* = 12), 65–79 years (*n* = 13), and 80+ years (*n* = 5). Nominal transcriptomic support was defined as an unadjusted endpoint or trend *p* < 0.05, and Benjamini–Hochberg false discovery rate (FDR) q values were reported in the supplementary transcriptomic summary. Because 14 genes were examined across multiple datasets and contrasts, nominal findings are interpreted as exploratory tissue context unless supported after FDR correction. Figure 2 summarizes supportive transcriptomic evidence for the direct sarcopenia and independent bulk-muscle contexts; locus-level support counts are integrated into Table 2.

Figure 2 summarizes supportive transcriptomic contextualization of shared sarcopenia-related loci.

### 3.6. Healthy Muscle Aging Context Extends Transcriptomic Support

**Figure 3:**
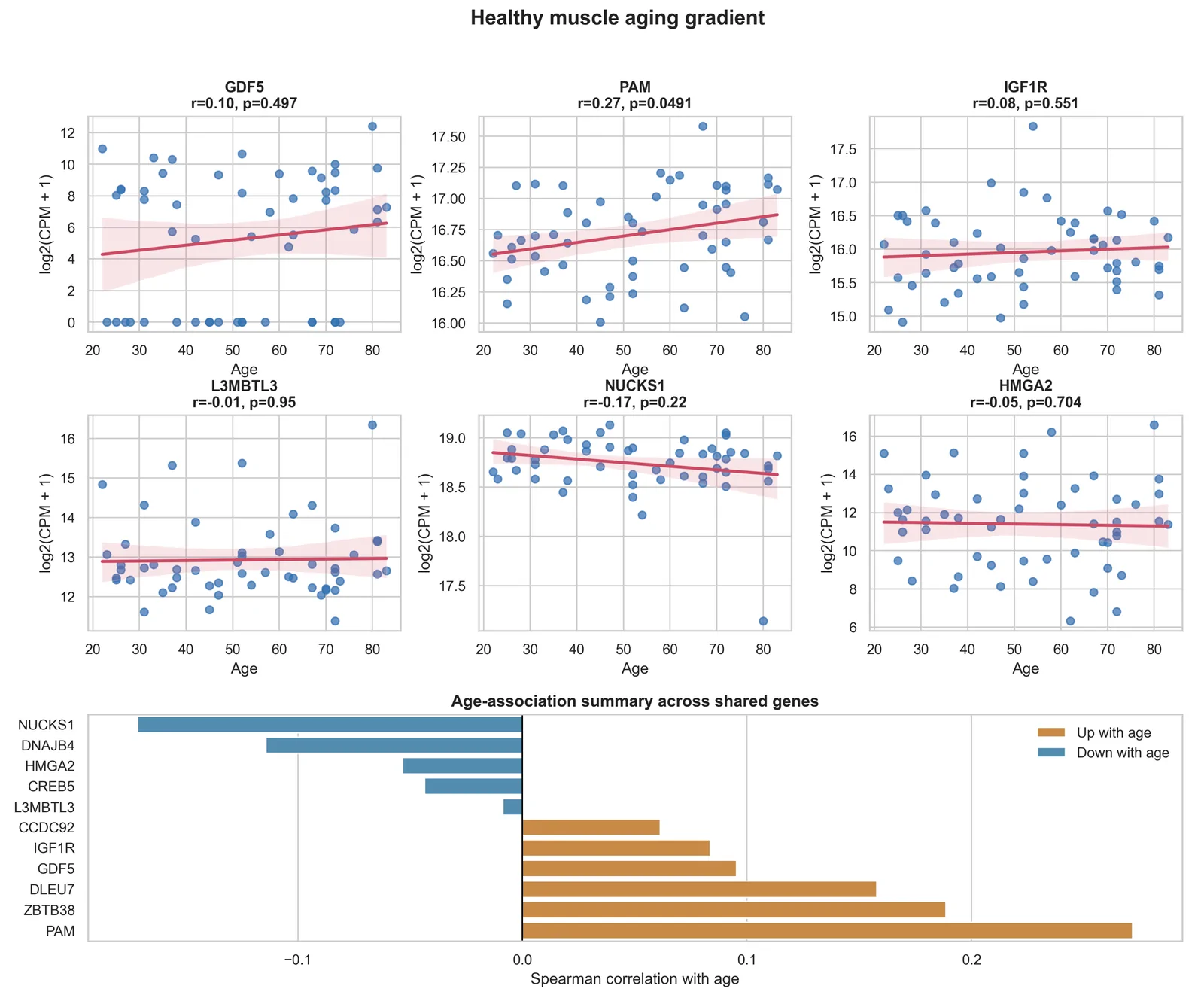
Healthy skeletal-muscle aging gradient of shared locus signals.Scatterplots display age-associated expression trajectories for selected overlap-derived genes across GSE164471 (*n* = 53). The solid red line represents the fitted linear regression trend, and the shaded band indicates the corresponding 95% confidence interval.; Spearman rho and nominal *p* values are shown for exploratory context. This dataset represents healthy skeletal muscle aging and was used to examine whether selected candidates exhibit age-related expression patterns outside a direct sarcopenia case–control setting.

The age-gradient dataset GSE164471 was presented as a distinct layer of evidence. Unlike the direct sarcopenia and case–control bulk-muscle datasets, this cohort traces healthy skeletal-muscle aging and evaluates whether overlap-derived signals remain detectable before overt sarcopenia classification. *PAM* was again notable in this setting, showing age-associated expression behavior consistent with a broader musculoskeletal-aging interpretation rather than a signal limited to a disease-labeled cohort (Figure 3).

### 3.7. Evidence-Guided Candidate List

**Figure 4:**
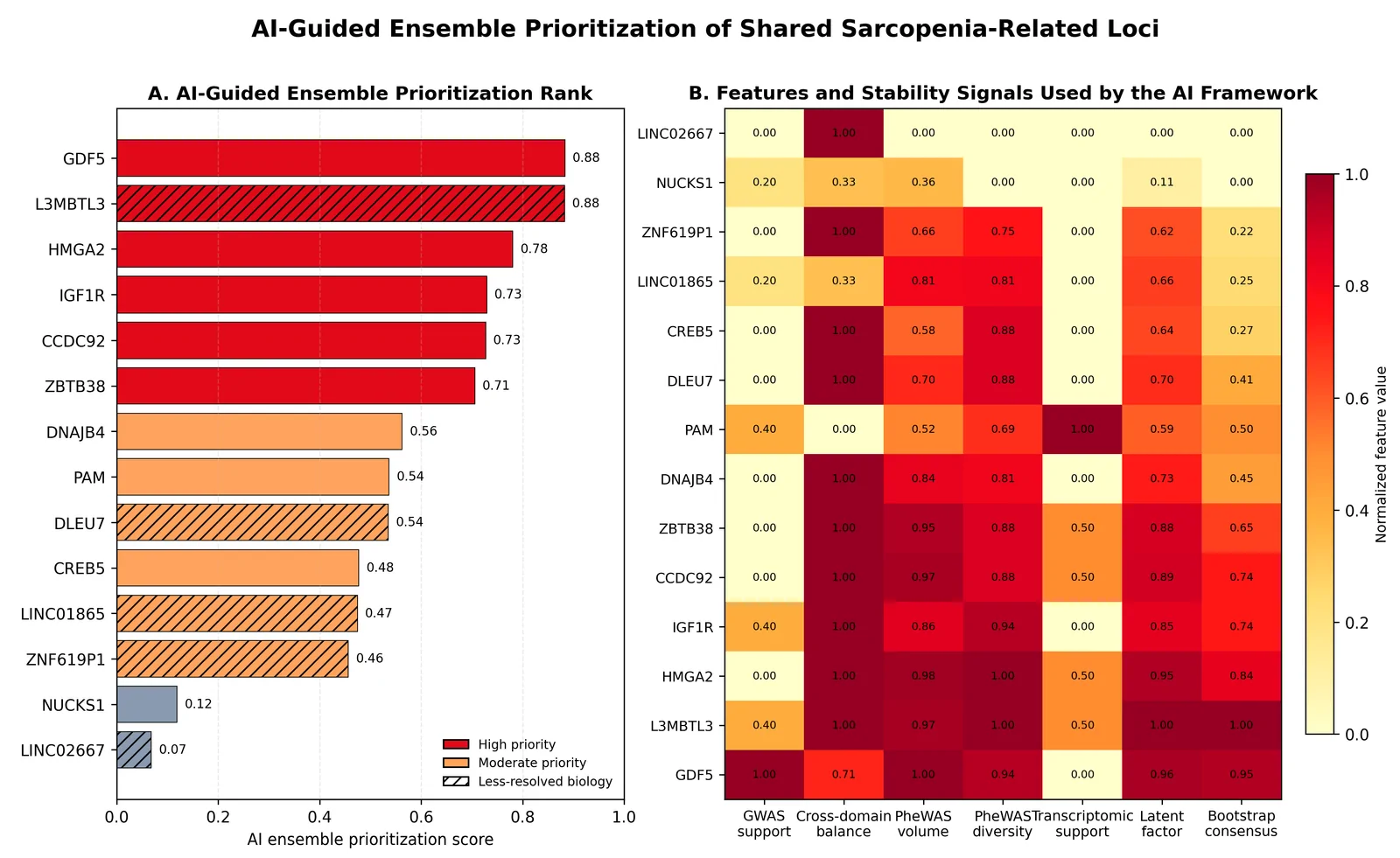
Multi-criteria evidence integration of shared candidate loci.Two-panel evidence-integration figure summarizing the secondary prioritization framework across the shared candidate loci. Panel (**A**) ranks catalog representative/mapped genes by an ensemble score derived from weighted evidence fusion, latent feature extraction, and Monte Carlo stability analysis across genome-wide association study (GWAS) recurrence, quantity-function balance, phenome-wide association study (PheWAS) breadth, and exploratory transcriptomic support; hatched bars indicate less-resolved biology genes, including *L3MBTL3*, *DLEU7*, *LINC01865*, *ZNF619P1*, and *LINC02667*. Panel (**B**) displays the normalized feature and stability matrix used for ranking. Gene labels are catalog mapped/reported-gene annotations and should not be interpreted as confirmed effector genes.

The evidence-integration framework ranked *GDF5* (ensemble score 0.8834), *L3MBTL3* (ensemble score 0.8826), and *HMGA2* (ensemble score 0.7807) as the highest-priority shared genes (Figure 4). *GDF5* and *L3MBTL3* therefore appear tied when rounded to two decimals, but the deterministic ranking used the unrounded scores. This ranking was driven by catalog recurrence, cross-trait balance, broad downstream PheWAS visibility, latent feature coherence, and rank stability. Weighting sensitivity showed that high-ranked loci remained influenced by catalog recurrence and PheWAS breadth, whereas removal of PheWAS breadth shifted ranking toward genes with stronger recurrence, balance, or transcriptomic support. The broader PheWAS evidence landscape, top downstream trait patterns, and sensitivity results are provided in the Supplementary Materials. High computational rank does not independently establish stronger mechanistic specificity. For *L3MBTL3*, the high rank appears to reflect balanced catalog evidence, broad PheWAS breadth, and rank stability; its biological relevance to muscle aging remains a hypothesis related to chromatin regulation and transcriptional silencing rather than a demonstrated sarcopenia mechanism.

Overall, the results support a layered, hypothesis-generating interpretation of sarcopenia-related proxy-trait catalog intersection. Exact-rsID overlap defined a candidate set, transcriptomic context provided exploratory tissue-level information, and multi-criteria evidence integration organized candidates for follow-up. This cumulative evidence structure supports transparent prioritization across complementary public data types, but it does not establish causal sharing, LD-level colocalization, or formal transcriptomic validation.

The less-resolved loci require particular caution. *L3MBTL3* encodes an MBT-domain methyl-lysine reader involved in chromatin-mediated transcriptional regulation, providing a plausible but indirect link to aging-related gene regulation rather than direct evidence of muscle-specific biology. *LINC01865*, *ZNF619P1*, and *LINC02667* are retained because they met the exact-rsID overlap rule, but their representative-gene labels may reflect proximity-based catalog annotation rather than confirmed effector genes. Broad PheWAS domain diversity may represent pleiotropic biology, shared aging mechanisms, or residual confounding from highly connected loci; it was therefore used only as a prioritization feature, not as proof of mechanistic centrality.

## 4. Discussion

This study shows that sarcopenia-related musculoskeletal aging can be examined through clinically aligned proxy-trait catalog intersection, but the present overlap should not be interpreted as a statistically validated multidimensional genetic model. Because sarcopenia is incompletely represented as a single phenotype in public association catalogs, its genetic context is more readily accessed through component traits including lean mass, grip strength, muscle weakness, frailty, and falling risk. By integrating these distributed signals, the present analysis generated a compact candidate locus set that may reflect parts of the multidimensional clinical definition of sarcopenia [1,2]. This framework provides a structured approach for developing hypotheses in public resources where the syndrome is not consistently indexed under one label.

The shared-locus pattern supports a focused, descriptive interpretation rather than a broad claim of a unified sarcopenia genetic architecture. The exact-rsID strategy increases auditability and specificity but may underestimate LD-mediated locus-level sharing and may be affected by overlapping source cohorts. Several contributing records derive from large biobanks, including UK Biobank, and some primary studies contribute data relevant to both muscle-quantity and function-frailty domains. The Supplementary Table S5 identifies source files with likely UK Biobank contribution or PMIDs appearing across both domains, and these entries should be considered at higher risk of participant-overlap inflation. The source studies also include predominantly European, TOPMed/multi-ancestry, Korean, Taiwanese, and COPD-specific cohorts, so population structure and ancestry-specific LD may affect whether an exact rsID represents the same underlying locus across comparisons. No dedicated long-range LD or population-stratification artifact-region filter was applied; therefore, broad PheWAS connectivity and exact-rsID sharing should be interpreted cautiously. The retained loci should be read as candidate hypotheses requiring harmonized summary-statistics analyses and external validation.

The transcriptomic context adds biological plausibility but does not validate the overlap-derived loci. *PAM* showed nominal support across both the independent sarcopenia cohort and the healthy-aging cohort, and *ZBTB38* and *CCDC92* showed nominal directional behavior in the independent cohort. The Supplementary Table S4 reports both raw *p* values and Benjamini–Hochberg FDR q values so that nominal and FDR-supported evidence can be distinguished. Because the transcriptomic datasets differ in cohort composition, age distribution, sarcopenia definition, platform, and statistical power, these findings are supportive and exploratory. The absence of FDR-confirmed transcriptomic signals for many genes should be considered when interpreting Table 2.

The multi-criteria evidence-integration layer adds an organizational component to this evidence structure. It does not replace the genetic overlap analysis or the transcriptomic contextualization; instead, it ranks retained candidates according to recurrence, quantity-function balance, pleiotropic breadth, transcriptomic support, latent-feature structure, and rank stability. This approach is most appropriately interpreted as transparent evidence integration rather than autonomous causal discovery. Within a hypothesis-generating study, such prioritization is useful for distinguishing loci supported by multiple complementary evidence layers from loci supported primarily by catalog breadth or pleiotropy.

The findings should be interpreted against phenotype-specific sarcopenia genetics rather than as independently validated shared architecture. Prior work has established GWAS signals for lean mass, grip strength, muscle weakness, frailty, falling risk, and cohort-specific sarcopenia definitions [11,12,13,14,15,16,17,18,19,20,21,22,23,24]. Additional sarcopenia-focused genetic literature, including aging-muscle genetic-association reviews, COPD-sarcopenia genetic analyses, WES/GWAS studies, exome-wide sarcopenic-obesity analyses, and systematic reviews of sarcopenia-related SNP or Mendelian-randomization evidence, provides closer context for the present proxy-trait design [33,34,35,36,37,40]. These references support the clinical and genetic plausibility of analyzing muscle quantity and function-related traits together, but they do not prove that the 14 exact-rsID overlap is causal, enriched, or mechanistically shared.

Several limitations should be considered when interpreting these findings. The analysis relies on grouped GWAS Catalog associations rather than a harmonized single-cohort sarcopenia GWAS, and therefore cannot resolve causal variants, fine-mapped sharing, ancestry-specific LD, or phenotype-specific mediation with the precision of full summary-statistics colocalization. The exact-rsID overlap strategy increases specificity but may underestimate broader genetic relatedness across domains. A +/−500 kb coordinate-window analysis was added only as an exploratory screen and should not be treated as LD-based colocalization. The trait-file permutation benchmark did not show that the observed 14-rsID overlap exceeded a catalog-level null distribution. Cohort overlap, especially among biobank-derived studies, may inflate apparent sharing. Gene assignment was based on GWAS Catalog mapped-gene and reported-gene fields rather than eQTL or variant-to-gene colocalization, leaving lncRNA, pseudogene, and intergenic loci less resolved. Transcriptomic evidence was exploratory and based on nominal unadjusted thresholds with FDR values reported for transparency. These limitations define the study as an integrative dry-lab screen and candidate-generation resource rather than a causal genetics analysis.

Future work could extend this framework by repeating the overlap analysis under alternative phenotyping rules, including explicit exclusion of falling-risk or COPD-proxy traits, incorporating harmonized summary statistics when available, and applying ancestry-aware LD clumping, colocalization, or fine-mapping approaches to prioritized loci. Independent skeletal-muscle cohorts and experimental systems will also be needed to evaluate whether the prioritized genes influence muscle quantity, strength, frailty-related vulnerability, or age-associated tissue remodeling. The present study therefore provides a traceable candidate architecture for prospective validation rather than a definitive mechanistic model.

## 5. Conclusions

In conclusion, this study supports the use of clinically aligned proxy-trait catalog intersection to generate sarcopenia-related genetic hypotheses in public resources. By integrating muscle-quantity and function-frailty GWAS domains, the analysis recovered a descriptive 14-rsID candidate intersection, but null benchmarking indicates that the overlap size should not be interpreted as statistically enriched sharing. The retained set was mainly grip/low-grip driven and does not establish causal, LD-level, or broad frailty/falls genetic sharing. Public skeletal-muscle transcriptomic context provided exploratory tissue context for selected loci, while the secondary evidence-integration layer provided a transparent method for organizing candidate follow-up. Together, these findings provide an interpretable starting point for future harmonized, ancestry-aware, and experimentally validated studies of sarcopenia-related genetics.

## Figures and Tables

**Table 1:** Summary of domain-level variant counts, shared overlap size, biological follow-up, and the secondary evidence-integration layer.

Study-Level Result	Summary
GWAS proxy traits merged	3 muscle-quantity files and 12 function-frailty files were integrated into two analysis domains.
Unique SNPs in muscle-quantity domain	1580
Unique SNPs in function-frailty domain	383
Shared SNPs between domains	14
Representative shared genes	14 loci summarized across 8 functional modules.
Highest-recurrence shared genes	*GDF5*, *IGF1R*, *L3MBTL3*, *PAM*, *LINC01865*
Public transcriptomic datasets interrogated	3 datasets: GSE226151, GSE167186, and GSE164471.
Genes with nominal transcriptomic support	5 genes; strongest signals included *L3MBTL3*, *HMGA2*, *CCDC92*, *ZBTB38*, *PAM*.
Broadest downstream PheWAS domains	Metabolic, Neurological, Immunological
Top AI-prioritized genes	*GDF5*, *L3MBTL3*, *HMGA2*

**Table 2:** Shared-locus table integrating evidence balance, transcriptomic support, downstream pleiotropic breadth, and evidence-integration prioritization rank.

AI Rank	Gene	Lead SNP	Functional Module	Quantity Evidence	Function/Frailty Evidence	Total Evidence	Transcriptomic Dataset Count	PheWAS Domain Count	AI Ensemble Score	Transcriptomic Support Note	Interpretability
1	*GDF5*	rs143384	Musculoskeletal development	3	4	7	0	23	0.88	No nominal signal retained	Mechanistically anchored
2	*L3MBTL3*	rs7740107	Other	2	2	4	1	24	0.88	GSE167186 sarcopenia-related down	Less resolved
3	*HMGA2*	rs10784502	Growth signaling	1	1	2	1	24	0.78	GSE167186 sarcopenia-related down	Mechanistically anchored
4	*IGF1R*	rs2871865	Growth signaling	2	2	4	0	23	0.73	No nominal signal retained	Mechanistically anchored
5	*CCDC92*	rs7301953	Metabolic regulation	1	1	2	1	22	0.73	GSE167186 sarcopenia-related down	Mechanistically anchored
6	*ZBTB38*	rs2871960	Transcriptional control	1	1	2	1	22	0.71	GSE167186 sarcopenia-related down	Mechanistically anchored
7	*DNAJB4*	rs34517439	Muscle contraction	1	1	2	0	21	0.56	No nominal signal retained	Mechanistically anchored
8	*PAM*	rs78408340	Neuromuscular signaling	3	1	4	2	19	0.54	GSE164471 age-related up; GSE167186 sarcopenia-related up	Mechanistically anchored
9	*DLEU7*	rs3118903	Other	1	1	2	0	22	0.54	No nominal signal retained	Less resolved
10	*CREB5*	rs62442203	Muscle development	1	1	2	0	22	0.48	No nominal signal retained	Mechanistically anchored
11	*LINC01865*	rs62106258	Other	2	1	3	0	21	0.47	No nominal signal retained	Less resolved
12	*ZNF619P1*	rs723149	Other	1	1	2	0	20	0.46	No nominal signal retained	Less resolved
13	*NUCKS1*	rs34305872	Transcriptional control	1	2	3	0	8	0.12	No nominal signal retained	Mechanistically anchored
14	*LINC02667*	rs372532055	Other	1	1	2	0	8	0.07	No nominal signal retained	Less resolved

Note: Gene labels in this table are catalog representative/mapped-gene annotations derived primarily from GWAS Catalog MAPPED_GENE and secondarily from REPORTED GENE(S). They should not be interpreted as confirmed effector genes without expression quantitative trait locus (eQTL), colocalization, or functional validation.

## Data Availability

All primary inputs used in this study were obtained from publicly available resources, including the NHGRI-EBI GWAS Catalog and public skeletal-muscle transcriptomic datasets. The archived GWAS Catalog trait files used for this analysis were stored locally with retained file timestamps on 15 April 2026; the exact official Catalog release version could not be independently reconstructed from the archived files. The public transcriptomic analyses used retained GEO count, TPM, SOFT, series-matrix, or processed summary files rather than FASTQ-level reprocessing. The R2 Supplementary Materials include the GWAS inventory, trait-grouping sensitivity table, evidence-integration weighting-sensitivity table, transcriptomic FDR summary, cohort-overlap risk table, coordinate-window overlap summary, reproducibility workflow note, and full reviewer-facing reproducibility script documenting how reviewer-facing tables were generated.

## Supplementary Materials

The following supporting information can be downloaded at https://www.mdpi.com/article/10.3390/life16081357/s1, Figure S1: PheWAS overview of shared sarcopenia-related loci; Figure S2: Top downstream PheWAS traits across shared loci; Table S1: raw GWAS inventory with GCST accessions and sample-size text; Table S2: trait-grouping sensitivity analysis; Table S3: evidence-integration weighting-sensitivity analysis; Table S4: transcriptomic raw *p* and Benjamini–Hochberg FDR summary; Table S5: cohort-overlap risk inventory; Table S6: exploratory +/−500 kb coordinate-window overlap summary, not LD-based overlap or colocalization; R2 reproducibility workflow note and full reviewer-facing reproducibility script.

## Abbreviations

The following abbreviations are used in this manuscript:

COPD	Chronic obstructive pulmonary disease
eQTL	Expression quantitative trait locus
EWGSOP	European Working Group on Sarcopenia in Older People
FDR	False discovery rate
FNIH	Foundation for the National Institutes of Health
GEO	Gene Expression Omnibus
GTEx	Genotype-Tissue Expression
GWAS	Genome-wide association study
LD	Linkage disequilibrium
NHGRI-EBI	National Human Genome Research Institute-European Bioinformatics Institute
PheWAS	Phenome-wide association study
SNP	Single-nucleotide polymorphism
SOFT	Simple Omnibus Format in Text
TPM	Transcripts per million
V2G	Variant-to-gene
